# The principal component analysis of key and significant features of the safety and nutritional value of Mahyaveh sauce

**DOI:** 10.1002/fsn3.3970

**Published:** 2024-01-23

**Authors:** Hoda Ghayoomi, Mohammad Reza Edalatian Dovom, Mohammad Bagher Habibi Najafi, Amir Pourfarzad

**Affiliations:** ^1^ Department of Food Science and Technology, Faculty of Agriculture Ferdowsi University of Mashhad Mashhad Iran; ^2^ Department of Food Science and Technology, Faculty of Agricultural Sciences University of Guilan Rasht Iran

**Keywords:** bacterial populations, biogenic amines, fermentation, fish sauce, physicochemical characteristics

## Abstract

The objective of present research is to evaluate the changes in the chemical, microbial, and biogenic amines in Persian fish sauce (Mahyaveh) during 40 days of fermentation. In the current survey, the parameters of salt percentage, pH, total nitrogen concentration, amino nitrogen concentration, Brix, color features, cadaverine, and histamine concentration were measured in the fish sauce. The amino nitrogen content, total protein, Brix, and salt were increased along with the progression of fermentation process. The microbial population of Mahyaveh sauce demonstrated that *lactic acid bacteria* (*LAB*), total bacterial count, and *Enterobacteriaceae* decreased during fermentation. The population of *lactic acid bacteria* and the total count of bacteria were around one logarithmic cycle lower in the presence of 10% salt than under low salt conditions. Histamine and cadaverine concentrations increased to 43.49 and 42.76 mg/kg during the fermentation period, respectively. As a result, the population density of histamine‐producing bacteria rose from 3.00 log CFU/mL at the beginning to 4.58 log CFU/mL at the end of process. The population density of cadaverine‐producing bacteria was 3.43 and 5.24 log CFU/mL on the 20th and 40th days of fermentation, respectively. Sensory evaluation results indicated that our sample of fish sauce had an overall acceptability score of 5.1 (good). On the other hand, Principal Component Analysis (PCA) demonstrated a positive correlation between the most of chemical parameters and the fermentation period. The concentration of cadaverine and histamine has a positive association with the pH and type of bacteria producing the biogenic amines.

## Graphical abstract

Iranian fish sauce was produced during the fermentation process (39°C, 40 days, and 12.65% salt). Physicochemical and microbiological analyses were carried out during the fermentation process. Finally, the association between different factors was examined using PCA.

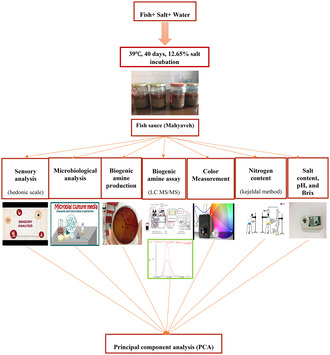

## 1 INTRODUCTION

Fish fermentation is a standard preservation method that depends on spontaneous fermentation (Fukami et al., 2004). In countries such as Korea (Mah et al., 2009), Thailand (Lopetcharat et al., 2001), Malaysia (Sim et al., 2015), and Egypt (Younis et al., 2010), fermented fish products are prevalent because of their long shelf life and unique flavor (Visessanguan et al., 2004). Fish sauce is a traditional high‐salt fermented fish frequently used as a food condiment in most Southeast Asian countries (Koo et al., 2016). Mahyaveh is a kind of fish sauce that is popular in the southern Provinces of Iran. It is usually prepared from salt‐fermented sardines or anchovies for 25–30 days (Zarei et al., 2012). Fish sauce is a clear brown liquid with a distinguished flavor and high nutritional value (Saisithi, 1994).

Proteolysis is one of the numerous biochemical processes that take place throughout fish sauce fermentation, and it is also considered one of the most contributors to fish sauce production (Klomklao et al., 2006). Numerous microorganisms are involved in fermentation processes and contribute to biochemical reactions (Lee et al., 2015; Smid & Kleerebezem, 2014). Among the microorganisms found in the fish sauce, *Streptococcus*, *Micrococcus*, *Bacillus*, and *Lactic Acid Bacteria* (*LAB*) are the most predominant genera (Guan et al., 2011; Zarei et al., 2012). Some of these microorganisms can hydrolyze proteins. Consequently, they produce peptides and free amino acids. As a result, these microorganisms can enhance the nutritive merit (Hiraga et al., 2005; Mizutani et al., 1992). Furthermore, LAB, which primarily contributes to produce lactic acid by fermenting readily accessible carbohydrates, is primarily responsible for fermented foods' flavor (Mokoena et al., 2016; Waters et al., 2015). However, some other microorganisms can cause harmful effects by producing compounds known as biogenic amines (Alvarez & Moreno‐Arribas, 2014; Fernández‐No et al., 2010).

Biogenic amines that are organic bases with low molecular weight (Gardini et al., 2016) are synthesized by the microbial amination or decarboxylation of certain free amino acids (Halász et al., 1994). Biogenic amines are involved in the immunological response, control of stomach pH and body temperature, and brain activity (Lorenzo et al., 2007; Sun et al., 2016). However, food consumption that is high in biogenic amines may lead to food intoxication. Hypertension, smooth muscle spasms, and impaired perception are all potential side effects of excessive intake of these amines (Hungerford, 2010).

The microbiota of fish sauce is considered a very crucial parameter in determining the distinctive qualities and characteristics of this product (Jung et al., 2018; Sun et al., 2016; Zhao et al., 2019). No quality control measures are applied to Mahyaveh fish sauce during traditional production of this product in southern Iran. Zarei et al. (2012) investigated the physical and chemical properties of 66 different types of local Mahyaveh. However, so far not many in‐depth studies have been performed on the relationship between Mahyaveh fish sauce's physicochemical characteristics and its microbial communities. The aim of this research was to assess the physicochemical characteristics, biogenic amines, and microbial properties of Mahyaveh during fermentation. Additionally, the relationship between physicochemical properties and the microbial community of Mahyaveh was also investigated.

## 2 MATERIALS AND METHODS

### 2.1 Materials

Dried anchovy fish was purchased from Bandar Abbas market. Packaged salt was obtained from the supermarket. AgNO3 (silver nitrate), KSCN (potassium thiocyanate), formalin solution (CH2O), sodium hydroxide (NaOH), and hydrochloric acid (HCl) were all purchased from Merck (Germany, German). Biogenic amine standards such as cadaverine (Cad) hydrochloride, histamine (His) dihydrochloride, were purchased from Sigma Aldrich (Buchs, Switzerland). HPLC‐grade Methanol and water were prepared from Carlo‐Erba (Sabadell, Barcelona, Spain). Bromocresol Purple (Bio Basic, Canada). Man Rogosa and Sharp (MRS), BHI, Violet Red Bile Glucose agar (VRBGA) (Ibresco, Italy), and Plate Count Agar (PCA) from (QUELAB, Canada).

### 2.2 Fish sauce production

To prepare Mahyaveh, 50 grams of dried anchovies were washed and rinsed. Then, the fish samples were put in glass jars containing brine (%12.65 salt). The containers were finally sealed and incubated for 40 days at 39°C. The mixture was blended daily throughout the fermentation process. The temperature, time of the fermentation process, and salt content of fish sauce were chosen based on the results of previous optimization experiments (Ghayoomi et al., 2023). To evaluate the chemical and microbial characteristics of Mahyaveh, they were sampled on days 5, 10, 20, 30, and 40. All trials were performed in three replicates. Sensory evaluation was performed on the finished product, after the filtrated brown liquid obtained on day‐ 40 of fermentation was mixed with roasted mustard, fennel, and pepper and put in glass containers. The glass containers were then exposed to sunshine for 15 days (Zarei et al., 2012).

### 2.3 Salt content, pH, and Brix analysis

Salt concentration in the samples was assessed using the Volhard procedure (AOAC, 2000). The salt content in the samples was calculated with the help of Equation 1. The pH of fish sauce was measured with a digital pH meter (86502, AZ, Taichung, Taiwan). The refractive index of soluble solids (Brix) was calculated using a refractometer (Pocket Pal‐3, ATAGO, Saitama, Japan).

(1) $$\text{Salt}\;\left(\%\right)=\frac{\left(V_{A}\times N_{A}\right)-\left(V_{B}\times N_{B}\right)\times 0.058\times 100}{W},$$

*V*_A_ = volume of AgNO_3_ (mL), *V*_B_ = volume of KSCN (mL), *N*_A_ = concentration of AgNO_3_ (N), *N*_B_ = concentration of KSCN (N), and *W* = weight of the sample (g).

### 2.4 Nitrogen content

The Kjeldahl technique was applied to define the total nitrogen concentration of fish sauce samples (AOAC, 2000). The total nitrogen content was computed as follows:

(2) $$\text{Total nitrogen content}\;\left(g/L\right)=\frac{\;\left(\left(V_{A}-V_{B}\right)\;N\times F\times 1000\right)}{S},$$

*V*_A_ and *V*_B_ = standard acid needed for the sample and blank in volume, *N* = normality of standardized acid, *F* = milliequivalent weight to nitrogen (14 mg/mmol), and *S* = Volume of sample digested (mL).

To measure amino nitrogen, the formalin titration procedure was employed (Lee et al., 2016). The fish sauce (10 mL) was mixed with 25 mL of water and the pH was adjusted with 1 N NaOH to 8 before the addition of 2 mL of 36% (w/v) formalin solution (CH_2_O). Then, 0.1 N NaOH was used to adjust the mixture's pH to 9.2. The amino nitrogen content of the samples was calculated with Equation 3.

(3) $$\text{Amino nitrogen content}\;\left(g/L\right)=\left(A-B\right)\times C\times 0.014\times D\times 1000/10,$$

where *A* is the titrated volume of 0.1 N NaOH consumed by the sample (mL), *B* is the titrated volume of 0.1 N NaOH consumed by the blank (mL), 0.014 represents the molar mass of the N (g/mmol), *C* is the concentration of NaOH solution, and *D* is the dilution factor, 1000/10 (to convert to liters).

### 2.5 Color measurement

The color characteristics of fish sauce were evaluated using L*, a*, and b* components. For imaging, fish sauce samples were located in a dark room at four 60 cm long, 10‐W fluorescent lamps. The lamps were fixed at a 45° angle and a distance of 45 cm from the sample. The distance between the camera and the sample was considered 25 cm. The pictures were taken with a digital camera (EOS 1000D, Canon, Tokyo, Japan). All of the images were taken using a 55‐mm zoom at a shutter speed of 1/3 of a second. Finally, they were saved in JPEG format. The images were then transformed into L*, a*, and b* space using ImageJ software version 1.48 v (Ghaitaranpour et al., 2018).

### 2.6 Biogenic amine assay

Two gram of the sample was picked up and centrifuged for 10 min at 15,871 × *g*. Then the supernatant phase was poured into another tube and it was diluted up to 5 mL. Then 250 mg of Dowex® 50 W X8 was added to the solution and it was vortexed for 1 min. The sorbent was separated by centrifugation. Then the analytes were eluted by HCl 0.1 M and a mobile phase (1:10, v/v).

Cadaverine and histamine analyses were done using LC–MS/MS on a chromatographic apparatus Alliance HT 2695 (Waters, Milford, MA, USA) coupled to a Waters Micro mass Quattro MS/MS spectrometer (triple quadrupole tandem mass spectrometry). Isocratic elution was performed by a mixture of methanol and water (pH = 3.5) (40:60). In all steps the flow rate of mobile phase was regulated at 0.2 mL/min. The analytes were separated on a Zorbax–SB–Aq C18 analytical column (Agilent Technologies, 100 mm × 4.6 mm, 3 μm particle size) held at 40°C. Multi‐reaction monitoring (MRM) mode with positive electrospray ionization was used for the MS/MS system. Desolvation and source temperature were regulated at 300 and 100°C, respectively. Mass Lynx (Version 4.1) software was used for data acquisition. Nebulizing and desolvation were done by nitrogen and argon, respectively. The characteristic molecular weight, Parent ion, daughter ions, cone voltage, and collision energy of histamine and cadaverine are presented in Table 1 (Mirzaei et al., 2022).

**TABLE 1:** MS/MS conditions in determination of the selected biogenic amines.

Analyte	Molecular weight	Parent ion (m/z) [M + H]^+^	Daughter ions (m/z)	Cone, voltage	Collision energy (eV)
Histamine	112	113.0	112, 95	24	30
Cadaverine	103	103.2	103, 86	27	30

### 2.7 Biogenic amine production

Using the Bover‐Cid and Holzapfel (1999) method, biogenic amine‐producing bacteria were enumerated. For this purpose, a BHI culture medium containing 1% of each precursor amino acid (L‐histidine mono hydrochloride and L‐lysine monohydrochloride) and 0.006% bromocresol purple was used. Then, all samples were incubated for 3 days at 37°C. The advent of a purple ring around the colony indicates a positive reaction. The culture medium without precursor amino acids was used as a control.

### 2.8 Microbiological analysis

The microorganisms in fish sauce have a significant impact on its quality. Under aseptic conditions, 1 mL of fish sauce was added to 9 mL of sterile saline (0.85% NaCl), and the mixture was homogenized for 1 min. The homogenized sample was then serially diluted to count the bacteria. To enumerate the total viable count, 1000 μL of fish sauce was cultured on Plate Count Agar (PCA) (QUELAB, Canada) and incubated at 30°C for 72 h. *Lactic acid bacteria* were grown anaerobically for 2 days at 37°C on Man Rogosa and Sharp (MRS) (Ibresco, Italy) agar medium. The count of *Enterobacteriaceae* was enumerated on Violet Red Bile Glucose agar (VRBGA) (Ibresco, Italy) after 24 h at 37°C. Plate Count Agar and MRS agar plates were prepared with 10% salt and incubated for 3–4 days at 37°C. Data were reported as log CFU/mL (Harrigan & McCance, 1976; Zeng et al., 2016).

### 2.9 Sensory analysis

The samples of fish sauce taken from 55 days' fermentation, as well as fish sauce from market, were assessed by 10 semi‐trained panelists according to Amerine et al. (2013) method. Mahyaveh sauce from Hormozgan and Fars Provinces, and the sauce prepared in our study were each coded with numbers 1, 2, and 3, respectively. The panelists in this evaluation were the students of the Ferdowsi University of Mashhad, who were both nonnative students and natives of the southern provinces of Iran. Using a 7‐point hedonic scale, panelists were asked to score the four aspects of odor, color, taste, and overall acceptability. On a 7‐point hedonic scale, a score of 1 indicates extremely inferior, a score of 4 indicates average, and a score of 7 indicates excellent. Fish sauce in the amount of 10 mL was put into a container and coded. Each sample was marked with a unique code and provided to the evaluators. Fish sauce was served with bread. The mouth was washed with water between each assessment.

### 2.10 Statistical analysis

All statistical analyses were done with the Minitab 17 software (Minitab Inc., State College, PA). The results were analyzed using a one‐way ANOVA. It was considered statistically significant at the *p* < .05 level. Data were presented as mean ± standard deviation (*n* = 6). There were two repeating batches of all fish sauce treatments. All parameters were evaluated in three replicates for each batch of fish sauces. Principal component analysis (PCA) was performed on chemical properties, biogenic amines, and microorganisms using Excel Stat 2016 software (Addinsoft, Inc.).

## 3 RESULTS AND DISCUSSIONS

### 3.1 Salt content

The production of fish sauce with reduced salt content was an objective of this study. In this study, the salt content of fish sauce increased as the fermentation time progressed (*p* < .05) (Table 2). During the fermentation process, the salt content rose from 12.59% to 12.95% (*p* < .05). Some other researchers also reported a rising salt trend (Hjalmarsson et al., 2007; Xu et al., 2008). According to Zarei et al. (2012), the salt concentration of Iranian fish sauce ranged from 7.4% to 17.1%. However, the concentration of fish sauce in Southeast Asian countries is reported to be between 15% and 20% (Sakpetch et al., 2022; Sim et al., 2015). Low concentrations of salt can improve the production process and product quality by increasing protein degradation, nutritional value, and fermentation rates (Xu et al., 2008). One of the key parameters in fish protease activity is the salt content in the environment, whereas pH and temperature can also have an impact on protease activity (Orejana & Liston, 1982).

**TABLE 2:** Effect of fermentation time on the physicochemical properties of fish sauce samples.

Fermentation, time (days)	Salt (%)	pH	Brix (%)	Total nitrogen (g/L)	Amino nitrogen (g/L)	a*	b*	L*
5	12.59 ± 0.16^B^	5.39 ± 0.05^D^	26.17 ± 0.03^D^	11.97 ± 0.09^E^	4.99 ± 0.16^C^	1.44 ± 0.01^E^	56.93 ± 0.09^A^	74.86 ± 0.07^A^
10	12.64 ± 0.02^B^	5.42 ± 0.03^D^	26.76 ± 0.03^C^	14.79 ± 0.09^D^	7.97 ± 0.12^B^	14.06 ± 0.07^D^	47.53 ± 0.17^B^	62.04 ± 0.07^B^
20	12.76 ± 0.09^AB^	5.90 ± 0.02^C^	26.80 ± 0.05^C^	15.88 ± 0.11^C^	8.11 ± 0.12^B^	17.52 ± 0.07^C^	43.38 ± 0.14^C^	57.98 ± 0.10^C^
30	12.83 ± 0.12^AB^	6.21 ± 0.01^B^	28.73 ± 0.08^B^	17.48 ± 0.16^B^	10.36 ± 0.12^A^	27.80 ± 0.13^B^	20.75 ± 0.10^D^	41.62 ± 0.11^D^
40	12.95 ± 0.24^A^	6.50 ± 0.03^A^	28.93 ± 0.12^A^	20.79 ± 0.14^A^	10.42 ± 0.19^A^	29.29 ± 0.09^A^	13.75 ± 0.10^E^	36.58 ± 0.08^E^

*Note*: Data are shown as the mean and ± SD of three replications. A, B, C, and other letters denote significantly different means at *p* < .05.

### 3.2 pH

The pH values of Mahyaveh samples are listed in Table 2. Significant differences were seen between the pH of Mahyaveh samples in the early and late stages of fermentation (*p* < .05). On the fifth day of fermentation, the pH of the fish sauce was 5.39; as fermentation time progressed, the pH went up to 6.50 (*p* < .05). The accumulation of alkaline compounds and the decomposition of amino acids from proteins and polypeptides lead to an increasing trend in pH with fermentation time (Duan et al., 2010; Ruiz‐Capillas & Moral, 2001). On the other hand, according to a report by Zaman et al. (2011), the pH of fish sauce at the start of fermentation was 5.42, and with the continuation of fermentation until the 120th day, the pH reached 7.35. Other researchers expressed a similar trend (Dissaraphong et al., 2006). According to investigations by Zarei et al. (2012), they reported that the pH of examined Mahyaveh samples ranged from 4.89 to 7.55. According to the CODEX guidelines, the fish sauce pH level must fall between 5.0 and 6.5 (Codex Alimentarius Commission, 2013).

### 3.3 Brix

The Brix degrees of Mahyaveh samples are presented in Table 2. The soluble solids (Brix) of Mahyaveh samples experienced an increasing trend with fermentation (*p* < .05). So the soluble solids content increased from around 26.17 on the fifth day of fermentation to 28.93 on the 40th day of fermentation (*p* < .05). Brix scale is a measure of how much protein has been hydrolyzed during fermentation (Lopetcharat & Park, 2002). The increase in brix of samples during the fermentation process can be attributed to the increase in the amount of free amino acids and small peptides released from the breakdown of fish proteins due to the presence of endogenous proteolytic enzymes and microbial enzymes (Beddows & Ardeshir, 1979). On days 0 and 60 of the fermenting process, the brix of fish sauce made from white fish from the Pacific Ocean was recorded as 29.2 and 39, respectively (Lopetcharat & Park, 2002). Additionally, Hjalmarsson et al. (2007) reported that the final brix of winter capelin fish sauce ranged from 19.2 to 22.4.

### 3.4 Total nitrogen

Table 2 displays the total nitrogen changes of the Mahyaveh samples. The total nitrogen content of these samples significantly increased from 11.97 on day 5 to 20.79 on day 40 of fermentation (*p* < .05). This result is in agreement with the results of Do Quynh Nguyen et al. (2021), who found that the duration of the fermentation can affect the total nitrogen concentration. According to the findings of Shim et al. (2021), the total nitrogen concentration of fish sauce produced from fresh anchovy fish was 1.32 and 1.81 mg/100 mL after 1 and 24 months of fermentation, respectively. The combined effects of fish muscle microbial degradation and autolysis could account for the increase in total nitrogen concentration (Kilinc et al., 2006). Total nitrogen is the quality index of fish sauce. Fish sauces classified as Grade 1 contain a total nitrogen content of more than 20 g/L, whereas the total nitrogen content of Grade 2 samples falls between 15 and 20 g/L (Thai Industrial Standard, 1983). Mahyaveh samples had more than 20 g/L of total nitrogen after 40 days of fermentation.

### 3.5 Amino nitrogen

Table 2 depicts the concentration of amino nitrogen in Mahyaveh samples during fermentation. The starting concentration of amino nitrogen was 4.99 g/L, and after 40 days of fermentation, it quickly increased to 10.42 g/L (*p* < .05). This result was in agreement with the findings of Zhu et al. (2021), who discovered the fermentation process significantly increased the amount of amino nitrogen in anchovy fish sauce. So, the concentration of amino nitrogen eventually reached 1.39 g/100 mL after 12 months of fermentation. Additionally, other researchers observed a similar trend (Do Quynh Nguyen et al., 2021; Ijong & Ohta, 1996; Klomklao et al., 2006). The breakdown of polypeptides by microbial and endogenous enzymes results in the formation of free amino acids and peptides with low molecular weight, both indicators of a rise in amino nitrogen concentration (Park et al., 2001). The Codex guideline states that amino nitrogen should not be less than 40% of total nitrogen (Codex Alimentarius Commission, 2013).

### 3.6 Color analysis

To control fish sauce color changes during fermentation, color values were established. The values of the fish sauce's color are exhibited in Table 2. The findings demonstrated that L* values presented a decreasing trend (*p* < .05). The samples' a* values increased during fermentation (from 1.44 to 29.29) (*p* < .05). The decline in the amount of L* and increase in the amount of a* causes the development of brown color in fish sauce. The brown color development is influenced by small peptides and free amino acids (Kawashima & Yamanaka, 1996). The b* values of the Mahyaveh samples significantly reduced from 56.93 to 13.75 during fermentation (*p* < .05). The change in hue may be caused by the presence or absence of molecules originating from fermentation activity and the Millard reaction (Izzo & Ho, 1992). According to Dissaraphong et al. (2006), L* and b* values decreased, whereas a* values increased over the course of 4 months of fermentation. Other researchers also confirmed that the L* levels in fish sauce decreased throughout the fermentation process (Klomklao et al., 2006).

### 3.7 Biogenic amines

**FIGURE 1:**
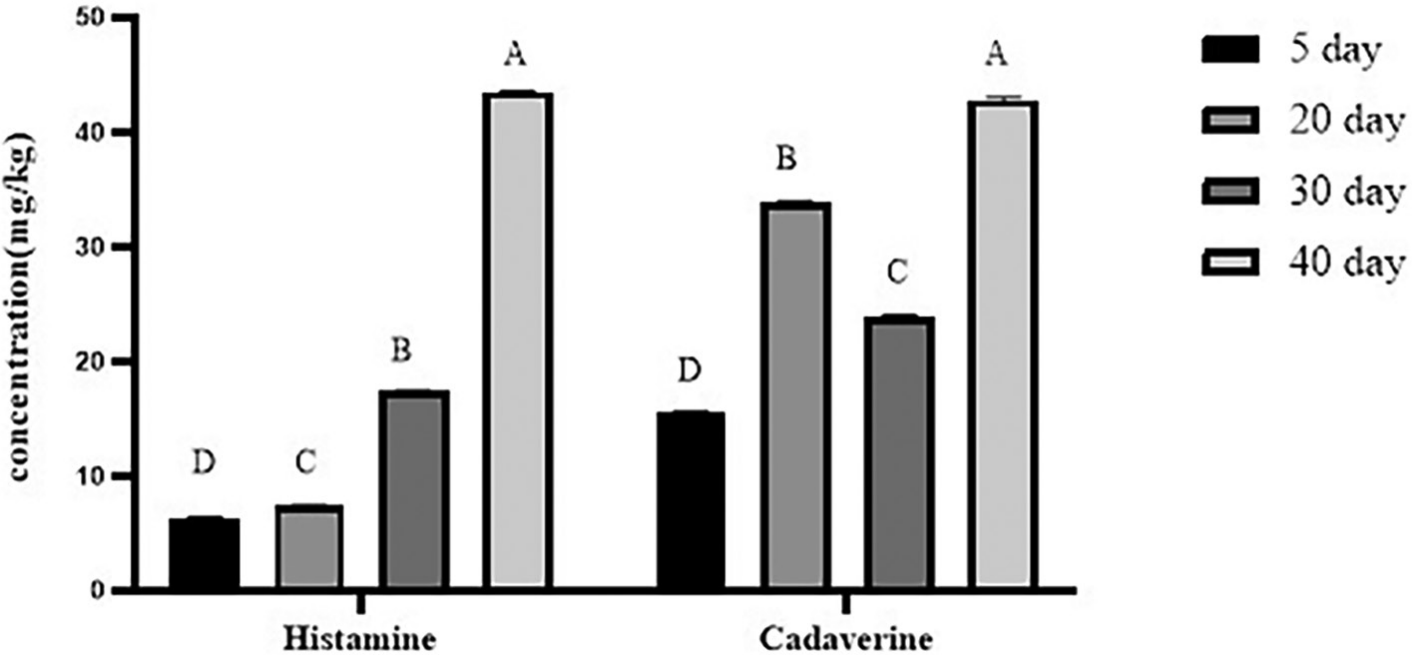
Changes in histamine and cadaverine content in Mahyaveh samples during fermentation. The standard deviation is represented by the bars. Significant differences are shown by different letters on the bars (*p* < .05).

Figure 1 presents the amount of biogenic amines (cadaverine and histamine) in Mahyaveh sauce. In the first 20 days of fermentation, the concentration of biogenic amines rose slowly, then increased rapidly until the 40th day of fermentation (*p* < .05) (Figure 1). Physiological toxicity and pathogenicity of histamine are dependent on the rate and extent of absorption. Biogenic amine excess intake can cause diarrhea and headaches (Rice et al., 1976). Histamine toxicity appeared to be enhanced when other biogenic amines, such as cadaverine, were present (Mah et al., 2002). The concentration of histamine was 6.25 mg/kg on day 5 of fermentation and rose to 7.31 mg/kg on day 20 (*p* < .05). The considerable increase in histamine during 20–40 days of fermentation (43.49 mg/kg) might be due to the favorable pH for microbial decarboxylase enzyme activity (Sun et al., 2018). Shim et al. (2021) stated that the histamine concentration in fish sauce rose considerably from the first to the third month of fermentation, which is in agreement with the results of this investigation. Zarei et al. (2012) stated that the histamine levels in 66 samples of Mahyaveh sauce ranged between 7916 mg/kg and no detection. Cho et al. (2015) found that during the 18‐month fermentation process, histamine levels increased in fish sauce.

Cadaverine is produced by the microbial decarboxylation of lysine. It can be used as a food health indicator (Chang & Chang, 2012). The level of this amino acid in Mahyaveh samples significantly increased from 15.554 mg/kg on the fifth day of fermentation to 42.763 mg/kg on the 40th day (*p* < .05) (Figure 1). The optimum pH for decarboxylase enzyme activity is about 6–6.5. Increasing biogenic amines concentration is directly affected by rising pH. Zaman et al. (2011) also outlined that the amount of cadaverine in fish sauce experienced an increasing trend as the process progressed. Thai researchers have stated that the concentrations of these two mentioned amino acids in Thai fish sauce are 45‐1220 (averagely 394±380 ppm) and Not Detected‐243 (averagely 89±51 ppm) mg/kg, respectively (Tsai et al., 2006).

### 3.8 Biogenic amines‐generating bacteria

**FIGURE 2:**
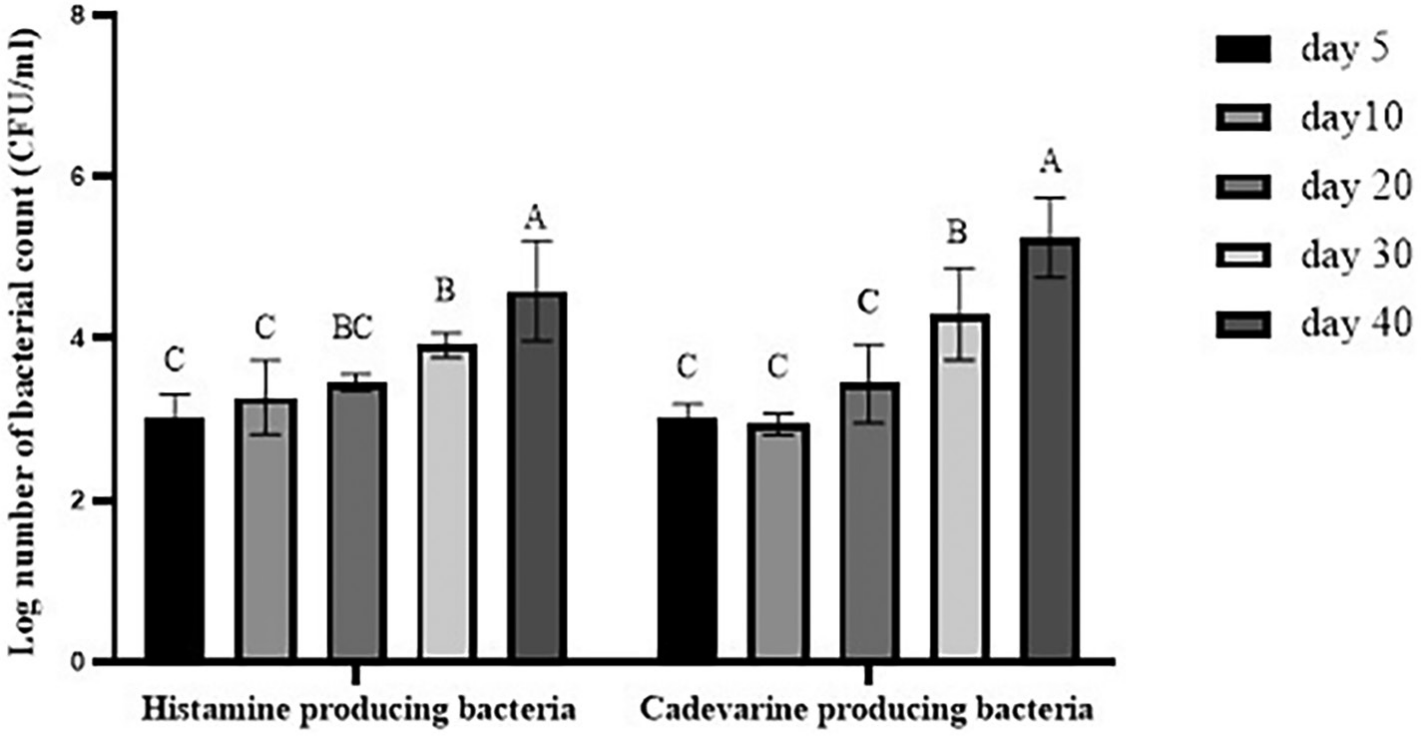
Histamine‐ and cadaverine‐producing bacteria concentration of fish sauce during fermentation. Significant differences are shown by different letters on the bars (*p* < .05).

The population changes of the microorganisms that generate biogenic amines are presented in Figure 2. As shown in Figure 2, there was 3.00 log CFU/mL of histamine‐producing bacteria at the beginning of fermentation. The number of above‐mentioned bacteria significantly showed growth continuously from day 20 until the fermentation's end (*p* < .05). At the end of fermentation, 4.58 log CFU/mL of these bacteria were enumerated. These findings were contradictory to those of Shim et al. (2021). During fermentation, they observed a decline in the number of aforementioned bacteria. However, in another study, it was found that these bacteria increased during fermentation so that the number of bacteria producing histamine rose from 4.55 log CFU/mL on the first day of fermentation to 5.72 log CFU/mL on the 20th day of fermentation (Zaman et al., 2011).

As depicted in Figure 2, from the first to the twentieth day of fermentation, no significant difference was seen in the number of cadaverine‐producing bacteria. However, a significant increase in these bacteria was seen on the 30th day of fermentation (4.30 log CFU/mL, *p* < .05). The number of cadaverine‐producing bacteria reached 5.24 log CFU/mL at the end of this process. According to Zaman et al. (2011), the number of bacteria that produce cadaverine grew from 4.96 log CFU/mL on the first day of fermentation to 5.78 log CFU/mL after 60 days of fermentation. Also, Sun et al. (2016) observed that during the course of 9 days of fermentation, the concentration of these two biogenic amines increased in fermented sausage. Biogenic amines are generated by bacterial decarboxylation of amino acids (ten Brink et al., 1990). The production of biogenic amines needs the presence of free amino acids, bacteria with decarboxylase ability, as well as favorable pH for bacterial growth and decarboxylase activity (Shim et al., 2021; Zaman et al., 2011). pH is an essential factor in the growth of bacteria‐producing biogenic amines (Lu et al., 2015). As demonstrated in this research, low pH values during the early days of fermentation reduced the growth of bacteria that are producing biogenic amines. Among the histamine‐producing bacteria which have been isolated from fish sauce *Tetragenococcus halophilus*, *Bacillus megaterium*, *Leuconostoc mesenteroides*, and other *LAB* strains were reported (Dapkevicius et al., 2000; Satomi et al., 2011; Tsai et al., 2006). On the other hand, *Enterobacter cloacae* and *Bacillus megaterium* are two of the bacteria that have been identified as bacteria that produce cadaverine in fermented fish (Song et al., 2010; Tsai et al., 2006). Therefore, it was revealed during our study that the count of bacteria that produce histamine significantly increased from the 30th day of fermentation (Figure 2). In the same direction, histamine concentrations have also elevated (Figure 1). It can be inferred from the findings regarding the count of bacteria that produce biogenic amines and changes in the amount of biogenic amines that the low level of biogenic amines observed at the beginning of fermentation may be due to low pH conditions that avoid the activity of decarboxylase enzyme (Sun et al., 2016).

### 3.9 Microbial analysis

**FIGURE 3:**
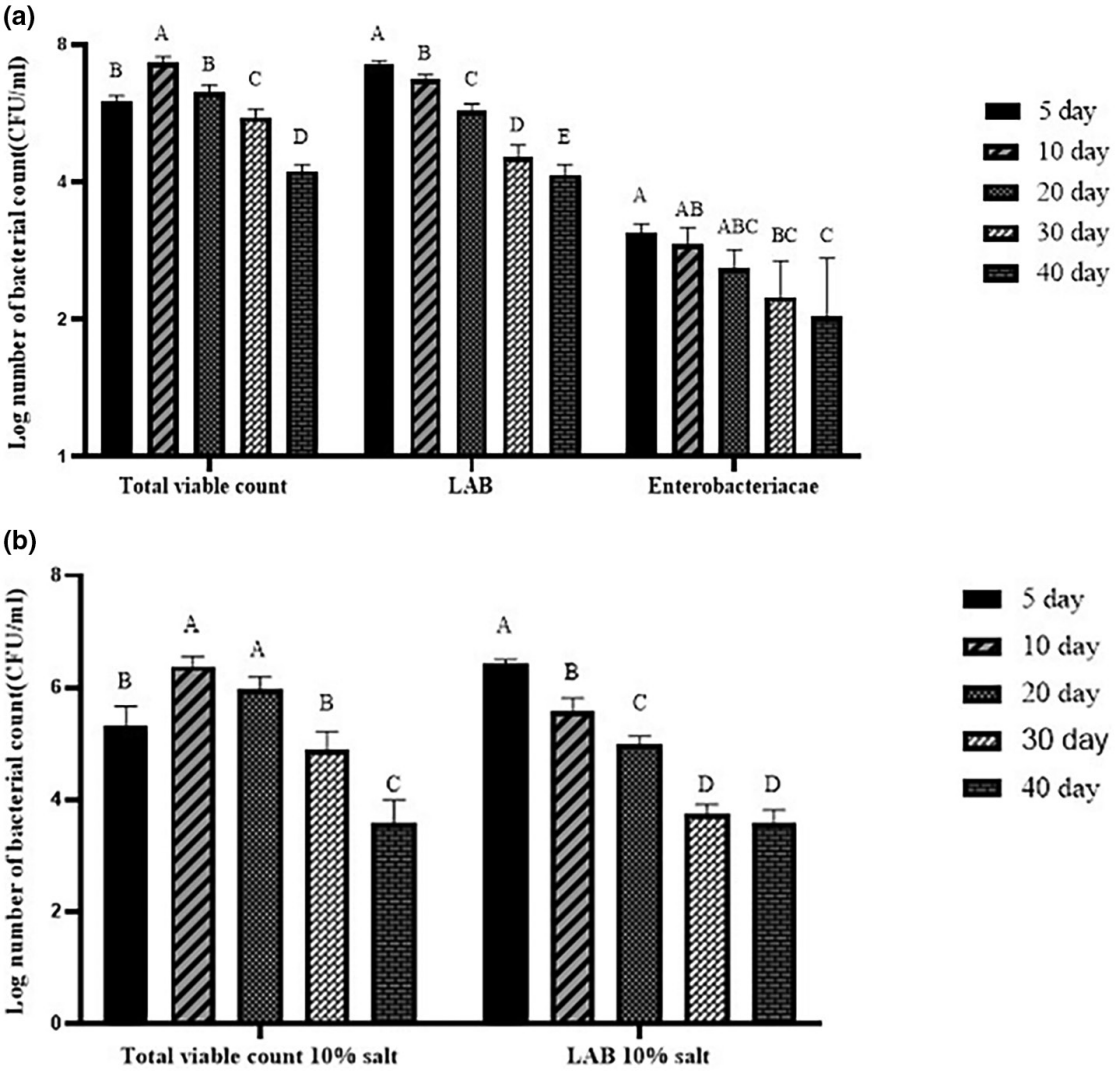
Changes in (a) total count bacteria, *lactic acid bacteria*, and *Enterobacteriaceae* counts (log CFU/mL), (b) total count bacteria + 10% salt, and *lactic acid bacteria* + 10% salt (log CFU/mL) in Mahyaveh sample during fermentation. The standard deviation is represented by the bars. Significant differences are shown by different letters on the bars (*p* < .05).

Figure 3 demonstrates the fluctuations in the microbial community of Mahyaveh. The initial total bacterial count was observed to be 6.03 log CFU/mL (Figure 3a). Then, it significantly rose until the 10th day of fermentation (7.29 log CFU/mL, *p* < .05). At the end of the fermentation, 4.22 log CFU/mL was recorded for total bacterial counts. Meanwhile, the total count of bacteria in samples with 10% salt is also shown in Figure 3b. It was found that the total count of bacteria initially rose until the 10th day of fermentation, then declined until the 40th day of fermentation (*p* < .05). The total count of bacteria was 5.30 log CFU/mL on the fifth day of fermentation and 6.37 log CFU/mL on the tenth day (*p* < .05). It was found that the total count of halophilic bacteria was around one log lower than the total count of nonhalophilic bacteria (*p* < .05). It demonstrates that salt inhibits the development and activity of microorganisms (Siringan et al., 2006). The total count of bacteria has increased in the early stages of process, perhaps because of favorable growth conditions and high water activity (Sun et al., 2016). Our findings are in line with those of Zaman et al. (2011), who found that the total aerobic bacterial concentration in fish sauce dropped throughout the fermenting process as a result of the decline in the water activity and the increase of the salt concentration.

On the contrary, Figure 3a shows that the number of *lactic acid bacteria* decreased throughout the process (*p* < .05). At the beginning of this process, their count was 7.24 log CFU/mL, and then declined to 4.14 log CFU/mL on the 40th day (*p* < .05). On the one hand, the growth trend of *LAB* was also investigated at a 10% salt concentration, and our findings are reported in Figure 3b. Based on the findings, salt was seen to inhibit and reduce the count of *LAB*. They also experienced a diminishing trend during the process (*p* < .05). The count of *LAB* at the final stage of fermentation with 10% salt was 3.59 log CFU/mL. The high count of *LAB* at the start of this process has led to the production of acid, which leads to a pH decline, which itself could be the cause for the low level of biogenic amines. As the fermentation process continues, the growth of these bacteria has reduced, most likely as a consequence of a decrease in water activity, the generation and accumulation of lactic acid metabolites, and other unfavorable conditions (Sun et al., 2018). Shim et al. (2021) stated that the number of *lactic acid bacteria* in a fish sauce made from 18‐h anchovy fish decreased from 14,438 to 164 copies. These outputs are in line with those of Singracha et al. (2017), who stated that the count of *LAB* in soy sauce declined from the start of fermentation to the final stage of this process. The total *LAB* count in Iranian fish sauce ranged between 0 and 5.54 log CFU/mL (Zarei et al., 2012).

During fermentation, the number of *Enterobacteriaceae* decreased significantly (Figure 3a, *p* < .05). The number of above‐mentioned was determined equal to 3.08 log CFU/mL on the fifth day of fermentation. No significant decrease in these bacteria was seen until the 20th day of this process. However, a considerable drop was noted from the 30th day through the end of fermentation (*p* < .05). On the 40th day of fermentation, the count of *Enterobacteriaceae* reached 2.03 log CFU/mL. *Enterobacteriaceae* are known to produce biogenic amines due to their significant decarboxylation activity of amino acids (Pircher et al., 2007). One of the reasons for the reduction of *Enterobacteriaceae* during fermentation could be related to unfavorable growth conditions and the bacteriocin generated by LAB (Lu et al., 2015). Similar to these findings, Sun et al. (2018) presented that the number of *Enterobacteriaceae* in fermented sausages reduced as fermentation progressed. On the one hand, Zarei et al. (2012) reported that Iranian Mahyaveh had an *Enterobacteriaceae* count ranging from 0 to 4.45 log CFU/mL.

### 3.10 Sensory analysis

Table 3 displays the taste, color, odor, and overall acceptability scores of Mahyaveh samples from Hormozgan (1), and Fars (2) Provinces, as well as the fish sauce sample obtained from our 55‐day fermented sample (3). Regarding color, the different Mahyaveh samples did not significantly differ from one another (*p* > .05). The sample of Hormozgan sauce obtained a lower score from the panelists in point of view of taste, smell, and overall acceptance (*p* < .05). However, no main difference was seen between the Mahyaveh sample from Fars Province and the sample of our study for odor and overall acceptance (*p* > .05). Overall acceptance of sample 3 was higher than the other two commercial samples (5.10). The Hormozgan sample obtained the lowest score regarding odor (3.5). It had a nasty odor. Based on the judgment of native and nonnative panelists, sample number 3 was scored as the best. The acceptance of sample 3 may be due to the adequate hydrolysis of protein, generation of free amino acids, and generation of flavor substances during fermentation. Thus, the organoleptic quality of fish sauce is highly affected by its chemical and microbial properties (Cai et al., 2015).

**TABLE 3:** Sensory changes in fermented fish sauce.

Treatment	Attributes			
	Color	Odor	Taste	Overall acceptability
Hormozgan sample	5.00 ± 1.24^A^	3.50 ± 1.60^B^	2.90 ± 1.44^B^	3.30 ± 1.25^B^
Fars sample	5.50 ± 0.97^A^	5.50 ± 0.97^A^	4.40 ± 1.17^A^	5.50 ± 1.27^A^
55‐day fermentation sample	4.80 ± 1.47^A^	5.60 ± 1.17^A^	4.70 ± 1.25^A^	5.10 ± 0.87^A^

*Note*: The average scores range from 1 (very poor) to 7 (extremely excellent). Significant differences (*p* < .05) are shown by different capital letter within the same column.

### 3.11 Principal component analysis

In occasions where a lot of variables complicate the observation, PCA can help identify the principal data structure, reduce it to a smaller group, and avoid multicollinearity in regression. The principal components matrix is the result of the eigenvector matrix based on the total variance. The goal of this method is to define and characterize the maximum variance with the smallest number of components (Habibi Najafi et al., 2019; Mahdavi‐Roshan et al., 2022; Pourfarzad & Taleb Derakhshan, 2021).

PCA as a multivariate symmetric approach represents the relationship between data sets. The goal is to explain as much of the total variance of the variables as possible, and the components are calculated as linear combinations of the original variables. Some authors have searched to correlate different properties of various products using PCA (Habibi Najafi et al., 2016; Manohar & Rao, 2002; Pourfarzad et al., 2012).

**FIGURE 4:**
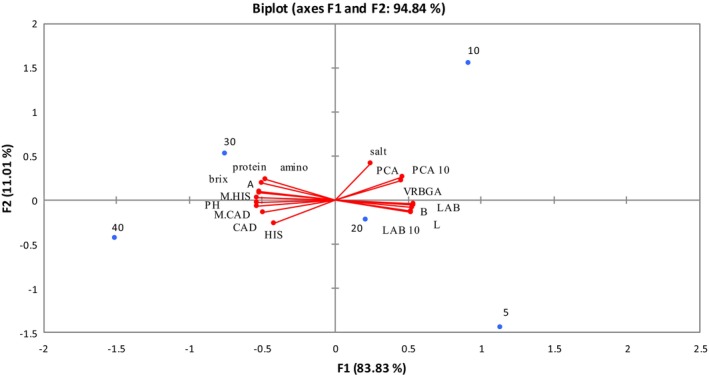
Principal component analysis of physicochemical parameters, histamine and cadaverine, and bacteria in Mahyaveh samples during fermentation.

The relationship among the physicochemical characteristics, biogenic amines, and microbial population of Mahyaveh sauce during the fermentation process was determined using PCA. The PCA revealed that the first two principal components were regarded as 94.84% of the total data variance (83.83% F1, 11.01% F2). Figure 4 demonstrates the positive correlation between biogenic amines, the biogenic amines‐producing bacteria, and pH. In addition, biogenic amines and pH have a positive relationship with amino nitrogen and Brix. Therefore, the increase in pH and the increase in the concentration of biogenic amines may be due to hydrolyzed proteins, and amino acids produced during fermentation. It has been determined that factors including pH, Brix, amino nitrogen, total nitrogen, and a* were positively correlated with fermentation times. So high values of these parameters are present in the samples on the last day of fermentation (Figure 4). Due to the high levels of amino nitrogen present during the last days of fermentation, fish sauce samples have a browner hue. Moreover, there is a negative association between *Enterobacteriaceae* (VRBGA) and the sample of day 40. They were positioned in two separate quadrants. This shows the low concentration of *Enterobacteriaceae* on the 40th day of fermentation. Also, this sample has a high level of safety in terms of safety quality.

Thus, systematic application of PCA can contribute to intensify and make stronger such comparisons. In addition, the application of PCA as a function of relevant empirical handwork (e.g., medical therapy, rehabilitation, or other interventions) can contribute to assess persuaded changes in coordination patterns. Identifying the components that are most impaired or most sensitive to treatment compared with healthy subjects could provide substantial and pivotal comprehension into the (mechanisms of) underlying impairment.

Reducing the salt content helps speed up the fermentation and reduce the duration of the process of fish sauce, while also improving the nutritional value of this product. According to the chemical and sensory findings, this product is of high quality in terms of taste and smell and does not have the problem of the unpleasant odor often found in commercial fish sauce.

Furthermore, the histamine level of this product is low, making it safe for high consumption without causing problems. These findings enable producers to continually improve their processes and products, ensuring the overall success and sustainability of the fish sauce industry in the food sector.

This product can serve as a basis for further studies on bioactive peptides due to its high protein content, which can be identified and used in the food and pharmaceutical industries.

## 4 CONCLUSIONS

Our research concentrated on the diversity of the bacterial community, biogenic amines, and chemical properties of Mahyaveh sauce during fermentation. Biogenic amines, bacterial populations, and some chemical characteristics have all been identified as significant parameters affecting safety and quality. During fermentation, it was observed that the bacterial population decreased. On the other hand, Mahyaveh sauce was found to have adequate levels of total and amino nitrogen. Regarding biogenic amines, the concentration of these compounds in Mahyaveh sauce during fermentation was observed to be lower than the allowable limit. The concentration of biogenic amines was positively related to the microorganisms that produced them and the pH. Hence, maintaining a low pH throughout the fermentation process might be an effective way to lower biogenic amines.

## AUTHOR CONTRIBUTIONS

**Hoda Ghayoomi:** Data curation (equal); formal analysis (equal); methodology (equal); writing – original draft (equal). **Mohammad Reza Edalatian Dovom:** Funding acquisition (equal); investigation (equal); project administration (equal); supervision (equal); writing – review and editing (equal). **Mohammad Bagher Habibi Najafi:** Funding acquisition (equal); investigation (equal); project administration (equal); supervision (equal); writing – review and editing (equal). **Amir Pourfarzad:** Data curation (equal); formal analysis (equal); methodology (equal); software (equal); writing – review and editing (equal).

## CONFLICT OF INTEREST STATEMENT

The authors declare no conflicts of interest.

## Data Availability

The data will be available from the corresponding author upon reasonable request.
